# Exploring Expectations, Ethical Dimensions, and Values for Voice-Enabled AI Assistants That Support Older Adults and Informal Family Caregivers: Participatory Speculative Design Study

**DOI:** 10.2196/79740

**Published:** 2026-06-16

**Authors:** Shweta Singh, Aqueasha Martin-Hammond

**Affiliations:** 1 Human-Centered Computing Department, Luddy School of Informatics, Computing, and Engineering, Indiana University Indianapolis, 535 W Michigan Street, IT 585, Indianapolis, IN, 46202, United States, 1 317-278-7686

**Keywords:** older adults, voice AI, voice assistants, ethics, design, human-computer interaction, participatory design, artificial intelligence

## Abstract

**Background:**

Continuous advancements in voice artificial intelligence technologies aim to assist older adults and caregivers, potentially improving quality of life and reducing caregiving burdens. Although research has explored the potential of voice-enabled artificial intelligence (VAI) assistants, such as Alexa (Amazon.com, Inc) and Google Home, to support older adults’ health in informal care settings, there remains a significant gap in understanding the ethical dimensions and values that may influence their future adoption by caregivers and care recipients.

**Objective:**

This research aims to explore older adult and informal family caregivers’ perspectives of VAI assistants for supporting informal care, including the ethical dimensions and values that influence their decisions about future adoption for these purposes.

**Methods:**

This research uses participatory speculative design to explore older adults’ and informal family caregivers’ perspectives of how VAI might support informal care in the future, and the ethical concerns they have about adopting VAI technologies. We conducted 8 workshop sessions with older adults and caregivers (n=9) over four months. Each phase focused on one of three goals: (1) to understand existing experiences, (2) to envision future VAI technologies, and (3) to reflect on ethical values that shape acceptance. In workshops, we aimed to gain insights into their experiences and challenges in managing informal care tasks and how future implementation of VAI might support the caregiving process to address their needs and concerns while emphasizing the ethical dimensions they value.

**Results:**

The findings suggest older adults and informal family caregivers see potential opportunities for VAIs to support informal aging care by automating daily health tasks to improve efficiency, enhancing mental health and well-being, and offering companionship. However, participants felt that VAI alone might not be sufficient to address the complex needs of informal care. Additionally, they raised several ethical concerns related to transparency, privacy, inclusiveness, trust, affordability, and autonomy, which they felt needed to be addressed to encourage adoption of VAI technologies for informal care in the future.

**Conclusions:**

Based on the findings, we offer insights and design implications for VAI systems that balance efficiency with ethical values to support diverse caregiving needs and potentially encourage future adoption in the informal care space.

## Introduction

### Background

Often unpaid, informal family caregivers play an important role in helping older adults age in place by assisting with activities of daily living and other health and wellness needs [1,2]. As more caregiving tasks move into the home, older adult-informal care relationships become increasingly important in supporting older adults in maintaining and achieving positive health outcomes [3]. At the same time, voice-enabled artificial intelligence (VAI) assistants (eg, Alexa or Siri [Apple Inc]), which leverage voice input/output and artificial intelligence (AI), are becoming increasingly prevalent in consumer homes and show promise for supporting older adults and their caregivers [1,2,4]. VAI assistants are generally helpful for addressing various accessibility needs among older adults [5-7]. However, research also indicates that older adults and informal caregivers see VAIs as potentially useful for supporting health care and other care needs at home [8-10]. For instance, VAIs have been found to be beneficial for informal caregivers and older adults with mild cognitive impairment [8], informal care dyads [10], and for scaffolding conversations between older adults and informal caregivers [9]. Thus, VAIs present a promising approach for enhancing the quality of life for older adults while helping to reduce some of the burdens faced by caregivers.

Significant prior research highlights the potential of VAIs to improve health care management and quality of life for older adults [10-12]. For older adult caregiving, research has also focused on understanding desired VAI features [4] and the benefits for tasks such as medication reminders, appointment scheduling [8,13], and improving communication between older adults and their informal caregivers [9,10]. Yet, ethical concerns such as privacy and transparency remain barriers to the acceptance and adoption of AI technologies such as VAIs generally [14,15], and these concerns are heightened within the health care context [16], where safety and confidentiality are paramount. Studies acknowledge that it is crucial to understand ethical considerations such as trust and transparency when considering AI-augmented health care interventions [17,18], calling for more emphasis on user values in health-related AI design and implementation [16]. However, little research has specifically explored the ethical values and dimensions that caregivers and older adults prioritize regarding VAI interactions in an informal caregiving context and how they might impact future adoption. Due to the diverse values individuals may have about AI use in health care [16], the growing ubiquity of VAIs for older adults’ health [10-12], and the importance of exploring values in different contexts [19], it is important to explore ethical values specific to the older adult informal caregiving context.

In this study, we use participatory speculative design to explore older adults and informal family caregivers’ ethical concerns about adopting VAI technologies. We aimed to gain insights into their experiences and challenges in managing informal care tasks, how future VAI features might support the caregiving process, and the ethical dimensions they value, including how those dimensions might impact potential acceptance. Based on our findings, we provide the following contributions: (1) We provide extended user perspectives on the role of VAIs in addressing informal caregiving needs, including the socioemotional aspects and burdens of informal care. (2) We highlight the ethical dimensions and values important to older adults and caregivers when considering accepting and adopting VAI assistants and the need to balance efficiency and ethical values in design to do good while doing no harm.

### Prior Work

We provide background on the importance of informal, unpaid caregiving for supporting older adults’ health and wellness needs, noting VAI assistants as emerging tools for supporting older adults and caregivers. We discuss open challenges of VAI acceptance and adoption for health and concerns raised in prior work that limit the adoption of VAI for health among older adults.

#### Informal Caregiving and VAI to Support Older Adults’ Care

Informal caregiving usually involves unpaid assistance with activities of daily living and other health tasks [2] by someone, usually a family member or a close friend, who is outside of the formal health care system [20]. As informal caregivers, family or close friends take on responsibilities of looking after another’s health and daily needs in addition to their own [2]. Informal caregiving tasks can include helping with feeding or personal hygiene, administering medication, accompanying care recipients to medical appointments, and fulfilling other essential needs in their daily lives [2]. For older adults, informal caregivers can play a significant role in maintaining their health when they are physically or mentally unable to perform daily tasks and self-care activities [3]. This form of caregiving can be physically and emotionally demanding, leading to exhaustion, neglecting one’s health, and financial strain among those who provide care [2,3,20]. Due to the demanding nature of caregiving for older adults, assessing and reducing the workload for these individuals overseeing their care is crucial.

One technology, VAI assistants, has emerged in recent years as a potentially accessible support for older adults and their caregivers. Recent studies [10-12,21-23] have suggested that including VAI assistants such as Alexa or Google Home within households has the potential to improve health care management and quality of life for older adults. There has also been an emerging focus on the potential of physiologically expressive voice-based conversational agents to deliver valuable social support in health care contexts [24,25]. In addition to these individual care tasks, researchers have also explored how VAI might support informal caregiving environments, focusing on supporting caregiving relationships and collaboration. Research has indicated that despite some open concerns, both hired (eg, paid) and family (unpaid) informal caregivers felt that VAI systems could benefit home care settings, including supporting tasks related to documentation, care coordination, and person-centered care [13]. Researchers have found that VAI assistants are viewed as potentially useful for supporting older adults and their caregiver dyads generally [10], as well as for supporting the care of those with mild cognitive impairment [8]. Rudnik et al [9] also found VAIs useful for helping older adults and caregivers scaffold care conversations. Therefore, there is evidence that older adults and caregivers feel VAI might help support informal caregiving environments. Yet open challenges exist.

#### Ethical Concerns in Voice Activated-Augmented Care

Some of the challenges of VAIs in health relate to unmet needs for desired interactions that are tailored to specific caregiving needs and tasks. For example, Corbett et al [10] found that while older adults and caregivers found existing voice assistants useful, they wanted additional features to help them with health and medical information. Zubatiy et al [8] found preferences for more assistance with activities of daily living. Therefore, while virtual assistants for older adults represent a notable step forward in developing socially adept conversational agents, there is a consensus that further refinement is necessary, especially in the health care context. Part of this refinement aims to ensure that these technologies are equipped to meet the demands of diverse real-world scenarios and align more closely with the user expectations and accessibility needs required to provide reliable interactions [11,22,24,26-28]. AI-enabled conversational health assistants such as VAIs have demonstrated their potential for accommodating individual preferences, particularly in situations where sensitivity plays a pivotal role, underscoring their relevance in health care and other contexts where tailored interactions are crucial [29]. However, while acknowledging these potential advantages, older adults and caregivers have also voiced ethical and trust concerns when leveraging AI-enabled health tools more generally [17], and these concerns have been found in VAI health research.

Ethics seeks to determine what is right, just, and morally acceptable within a given context or society [14]. Ethics in AI is a complex and evolving field, with convergence around 5 key principles: transparency, justice and fairness, trust, responsibility, and privacy [14]. Examining ethical components and their value when designing AI systems is thought to be the foundation of any successful interaction between humans and AI systems. VAI assistants have been found to raise ethical concerns with users [15,19,30-32]. The systematic examination of ethical concerns regarding voice assistants by Seymour et al [19] (eg, Amazon Alexa and Google Home) more broadly found that privacy was the most cited concern, yet other concerns related to accessibility, social interaction (eg, anthropomorphism and emotional connection to machines), and social order (eg, impact on parent-child relationships) were also discussed. Similar concerns apply to VAIs used within a health care context; however, examining ethical values is particularly important due to health care’s safety-critical and privacy-sensitive nature [33]. For example, research has shown that the degree of health information sensitivity can impact users’ preferences for interactions with VAI assistants such as Google Home [34]. Patients tend to prioritize trustworthiness and transparency when engaging with health-related assistants [21], and different aspects of trust, such as credibility and service quality, have also been found to impact perceptions of health-related VAIs and users’ intentions to use them [33]. Therefore, though AI-driven assistive and voice-based interfaces offer substantial benefits, studies suggest an imperative need for tailored approaches that prioritize differences in context and individual and group experiences to establish trust and ethical standards [17], including exploring participatory methods to democratize design, expand who is included in the design, and prioritize usefulness [33,35].

## Methods

### Study Design

**Figure 1.**
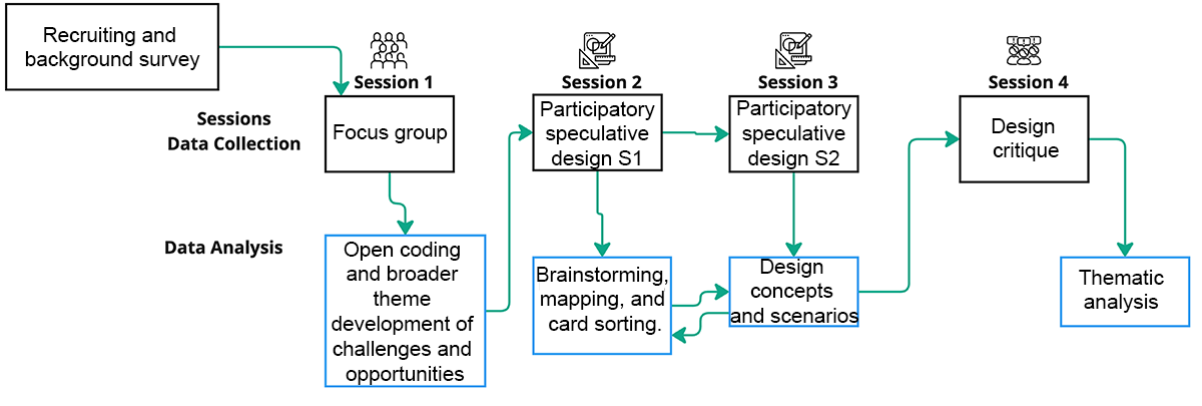
Image of the participatory speculative design process. The process included 4 workshop sessions, including a focus group, two participatory speculative design sessions, and one design critique session.

We conducted a 3-phase study (Figure 1) using participatory speculative design to explore how future VAI assistants might support informal caregiving practices among older adults and caregivers, as well as the values influencing the potential adoption of these technologies. Each phase addressed one of three goals: (1) understanding existing experiences, (2) envisioning future VAI technologies, and (3) reflecting on ethical values that shape acceptance. This study focused on the following research questions (RQs): (RQ1) How do participants perceive the role of VAIs in supporting caregiving tasks? (RQ2) What values do caregivers and older adults have around VAI assistants that support day-to-day care tasks? (RQ3) What ethical concerns do caregivers and older adults have about VAI assistants that support day-to-day care tasks?

### Ethical Considerations

The Institutional Review Board at Indiana University reviewed and approved this study as exempt (Institutional Review Board application #21351) before recruitment or data collection began. In the first workshop session (session #1), we provided participants with a printed study information sheet that explained this study, the data we would collect, their rights as participants, and our procedures for minimizing risks to privacy and confidentiality. We allowed time for participants to read the sheet and also explained the information verbally. This study’s information sheet included details on payment procedures (US $35 per session completed), principal investigator contact information, and how to contact our Institutional Review Board office. Participants were also informed of efforts to keep their personal information confidential, including restricting access to their data to only the research team and destroying identifiable data (such as audio or video recordings) at the end of this study. They were also told that their data might be shared with the University or the federal agency funding the research, if required by law. Participants were informed of their right to withdraw from this study at any time. In accordance with our university’s procedures for exempt research, participants provided verbal consent to continue after reviewing and asking questions about this study’s information sheet.

### Rationale for Participatory Speculative Design

While voice-activated technologies such as Siri and Alexa are currently available to consumers, they are often designed primarily for non–health-related uses. Therefore, while the current study of these tools for health is growing as an emerging technology, they are not commonly leveraged in health settings due to open concerns about safety [36] and privacy, and are typically not widely available outside of a research context. We chose speculative participatory design as an approach because it provided the flexibility for participants to imagine a future for these technologies in a health context, specifically for supporting informal, aging care, and to reflect on the ethical considerations around future adoption and integration within the home and informal care environment.

Speculative design offers users a way to speculate about future AI technologies and enables researchers to analyze those speculations to uncover values [37], which is a powerful approach for exploring ethics in the rapidly developing field of AI design [38]. The proposed method intersects with other human-computer interaction (HCI) methods, including participatory design [39], value-sensitive design [40], and design fiction [37]. While each of these approaches has been found useful for designing AI systems [21,41], speculative approaches have been useful for helping users envision future AI systems and the corresponding values that may not yet exist, or for imagining possible new futures [31,37,42-44]. Formally, speculative design often leverages the collective strengths of each method to use fictional stories to “collectively envision, design, and critique future AI agents,” thereby allowing researchers to understand how people imagine future technologies and the values they consider important when designing them [37]. As an approach, it allows for the imaginative exploration of “future configurations of technology and society within the world” by adopting both creative and critical perspectives, including critiquing existing AI practices [38,45].

### Recruitment and Participants

We recruited 9 participants, including 3 older adults and 6 caregivers, for a 4-month study (January-April 2024). The criteria for caregiver participation were that individuals be 18 years of age or older and serve as an informal, unpaid, family or friend caregiver to an individual 65 years of age or older. The criteria for older adult participation were that the individual be 65 years of age or older and be the recipient of informal (eg, family or friend), unpaid care. Additionally, because of our goal of speculative co-design of VAI technologies, we required that participants be currently using or have an interest in potentially adopting or using these technologies in an informal care setting in the future.

Our recruitment method was purposeful, aligning with our need for participants with specific caregiving experiences and interests in using and designing VAI assistants for that space. During recruitment, we found that 4 of 6 of our caregiving participants also self-identified as older adults. In those cases, we provided participants the flexibility to choose which role they would like to take on for the workshop sessions, letting them know that they could also share their perspectives as older adults receiving care. However, we did experience participant dropout over the 4 months. A total of 7 participants attended all 4 sessions. Further, 1 caregiver attended only 2 sessions in their series, and 1 caregiver attended only 3 sessions. Participants did not continue due to busy schedules and other time commitments (Table 1).

**Table 1.** Participant demographics and participation in study sessions.

Participant	Age group (years)	Sex	Caregiver or older adult	Sessions participated
P1	35‐45	Female	Caregiver	S1, S2, S3, S4
P2	55‐65	Female	Caregiver	S1, S2, S3, S4
P3	65‐75	Male	Caregiver	S1, S2, S3, S4
P4	65‐75	Female	Caregiver	S1, S2
P5	65‐75	Female	Caregiver	S1, S2, S3
P6	65‐75	Female	Caregiver	S1, S2, S3, S4
P7	75‐85	Female	Older adult	S1, S2, S3, S4
P8	75‐85	Male	Older adult	S1, S2, S3, S4
P9	75‐85	Female	Older adult	S1, S2, S3, S4

Informal caregiver participants served primarily as family caregivers, including spouses, children, and siblings of the care recipient (Table 1). Their ages ranged from 35 to 75 years, with the majority also self-identifying as older adults (65-75 years). Most caregivers were primary caregivers (ie, the responsible party for providing care), although some occasionally received additional help from others. Caregiver participants supported individuals with a wide range of conditions, including dementia, chronic diseases, amputations, and cancer. Their caregiving durations spanned from 6 months to more than 10 years. The type of care tasks they assisted with involved primarily instrumental activities of daily living (eg, housework, transportation, and managing finances), companionship and emotional support, routine activities of daily living (eg, bathing, toileting, and eating), and medical and nursing tasks, such as injections and colostomy care.

Older adult participants ranged from 75 to 85 years of age and had children or spouses as primary caregivers. They had from 2 to 5 doctors and nurses on their health care team in addition to 1‐2 other caregivers who help them out with additional health tasks either daily or a few times a year. Older adults and caregivers were familiar to very familiar with voice assistants such as Siri and Alexa.

### Participatory Design Sessions

#### Overview

Each group of participants (older adults and caregivers) engaged in 4 workshop sessions over the 4 months (Figure 1). Therefore, we conducted 8 group workshops in total, 4 with informal caregivers and 4 with older adults who receive care from family or friends. Topics and session progression were similar for both groups. Workshops were conducted online and in person. Initially, workshops were planned for in person; however, to accommodate participants with mobility and other challenges that prevented them from attending in person, we held some sessions online. During in-person workshops, participants engaged in activities such as card sorting, collaborative sketching, and crafting scenarios through role-play and discussion (Figure S2 in Multimedia Appendix 1). In-person and online workshops were conducted similarly, using online tools including videoconferencing software with breakout rooms and online collaboration tools (eg, Miro) to pivot and replicate activities for online participants. Each workshop session lasted approximately 1‐1.5 hours (60‐90 min), and participants were paid US $35 per session.

#### Session 1: Understanding Current Informal Caregiving Experiences

In the first workshop session, we aimed to create a common foundation for envisioning future technologies by understanding participants’ experiences with caregiving, AI, and VAI technologies. We sought to gain a comprehensive understanding of the dynamics of informal family caregiving by engaging both caregivers and care recipients in an initial focus group to explore experiences shaping their caregiving values. We asked them to describe their daily routines, experiences, challenges, and key areas for improvement.

We also introduced AI and VAI assistants, sharing definitions of AI, its capabilities, and common VAI scenarios. We provided examples of consumer-facing VAI assistants, such as Siri, Alexa, and Google Assistant, and facilitated open discussions to address questions and assess participants’ existing knowledge. We noted key points about experiences or challenges with caregiving or VAIs on sticky notes to prepare for session 2. The focus groups lasted approximately 60 minutes.

#### Sessions 2 and 3: Imagining VAI Informal Caregiving Futures

The second and third sessions aimed to envision VAI assistants that support informal caregiving. We used participatory speculative design to conceptualize AI voice applications that participants believed could help them in informal caregiving settings (Figure 1). In session 2, the group reviewed and validated notes from session 1 about participants’ caregiving experiences and concerns. Participants used affinity mapping and brainstorming to identify and prioritize how VAI could support their informal caregiving experiences. To help prioritize initial ideas for further discussion, participants used card sorting to rank ideas and reflected as a group on (1) the potential feasibility of each idea and (2) how it might meet their needs.

After brainstorming and card-sorting, we used prompts, as shown in Figure S1 in Multimedia Appendix 1, to engage participants in co-design activities, such as scenario-based role-playing. Using these prompts, they brainstormed and created concepts for VAI interfaces suited for informal caregiving. During the third session, we continued these activities to complete the design concepts by creating a final set of scenarios. Consistent with the principles of speculative design [37], our goal was to explore values through the design of future technologies within the context of participants’ narratives of use. While emphasizing VAI applications to keep discussions grounded in participants’ stories of future use, sessions 2‐3 also aimed to understand why participants made specific design choices, thereby enhancing our insight into their ethical values. Each workshop lasted about 90 minutes.

#### Session 4: Ethical Critiques of VAI for Informal Care

In the fourth session, we asked participants to revisit the concepts and scenarios they generated in sessions 2‐3 to reflect more deeply on their ethical values. Some ethical values emerged naturally in sessions 2‐3 as participants discussed ideas; however, in session 4, we asked participants to deliberately critique their scenarios through the lens of different ethical dimensions (eg, privacy, transparency, trust, and user autonomy) [14], consider potential challenges that could restrict their adoption, and discuss any needs related to various ethical dimensions, whether positive or negative. We also encouraged them to discuss additional issues that might not have been listed, using their own words, which we later categorized during analysis. The workshop lasted approximately 90 minutes.

### Data Analysis

Each focus group and workshop session lasted 60‐90 minutes. All sessions were recorded with the participants’ permission. Data included transcripts generated from over 660 minutes of audio recordings from 8 workshops (4 with older adults and 4 with caregivers), researcher notes, and physical artifacts. While we did experience dropout during recruitment, resulting in a smaller sample, we observed recurring themes (thematic saturation) [46]. We used thematic analysis [47] to analyze the data. To conduct the analysis, the first author reviewed transcripts using memos to highlight interesting aspects of the data. Using a reflexive thematic process [47], we then used inductive and deductive coding to identify recurring themes from the data, focusing both within and across participant groups. Inductive coding was initially used to identify codes related to questions about caregiving experiences, opinions about VAIs, and discussions of ethical concerns. We later used deductive coding to further categorize ethical concerns into existing ethical dimensions: transparency, trust, privacy, justice and fairness, and freedom and autonomy (Table S1 in Multimedia Appendix 1), aligning with known AI ethical dimensions identified in prior literature [14]. We met iteratively [48] to synthesize data, discussing and categorizing codes into themes. We used an axial coding process to organize the identified codes into broader categories. Analysis of data from participatory design activities, such as lists of caregiving challenges and VAI ideas, was taken directly from data generated during those activities and was refined by the research team using affinity diagramming to merge similar themes across workshops. Three high-level themes emerged: (1) caregiving experiences, (2) opinions about VAIs for caregiving, and (3) ethical dimensions and values aligning with study goals. A detailed summary of themes and subcodes is included in Table S2 in Multimedia Appendix 1.

## Results

### Overview

Findings from this study shed light on the complex interplay of emotional, physical, and logistical difficulties experienced by older adults and caregivers when engaging in caregiving tasks, as well as their needs and challenges, and their ideas for how VAI might support informal care in the future. Our findings also highlight tensions regarding the potential benefits of VAI and ethical concerns associated with its adoption in a care environment.

### Envisioning VAI for Informal Care Support

#### Caregiving Experiences

From session 1, we learned that caregivers and care recipients represented varied caregiving experiences. However, some caregivers and care recipients have overlapping experiences of care. Aligned with HCI work exploring technology experiences of smaller numbers of participants [49], we present these experiences as personas. Our personas were based on real data and represent a grouping of common experiences across different participants. This method was used in the study by Waycott et al [49] exploring nontech use, where participant numbers were low (10 total) to balance sharing data about experiences while respecting participant confidentiality.

**Figure 2.**
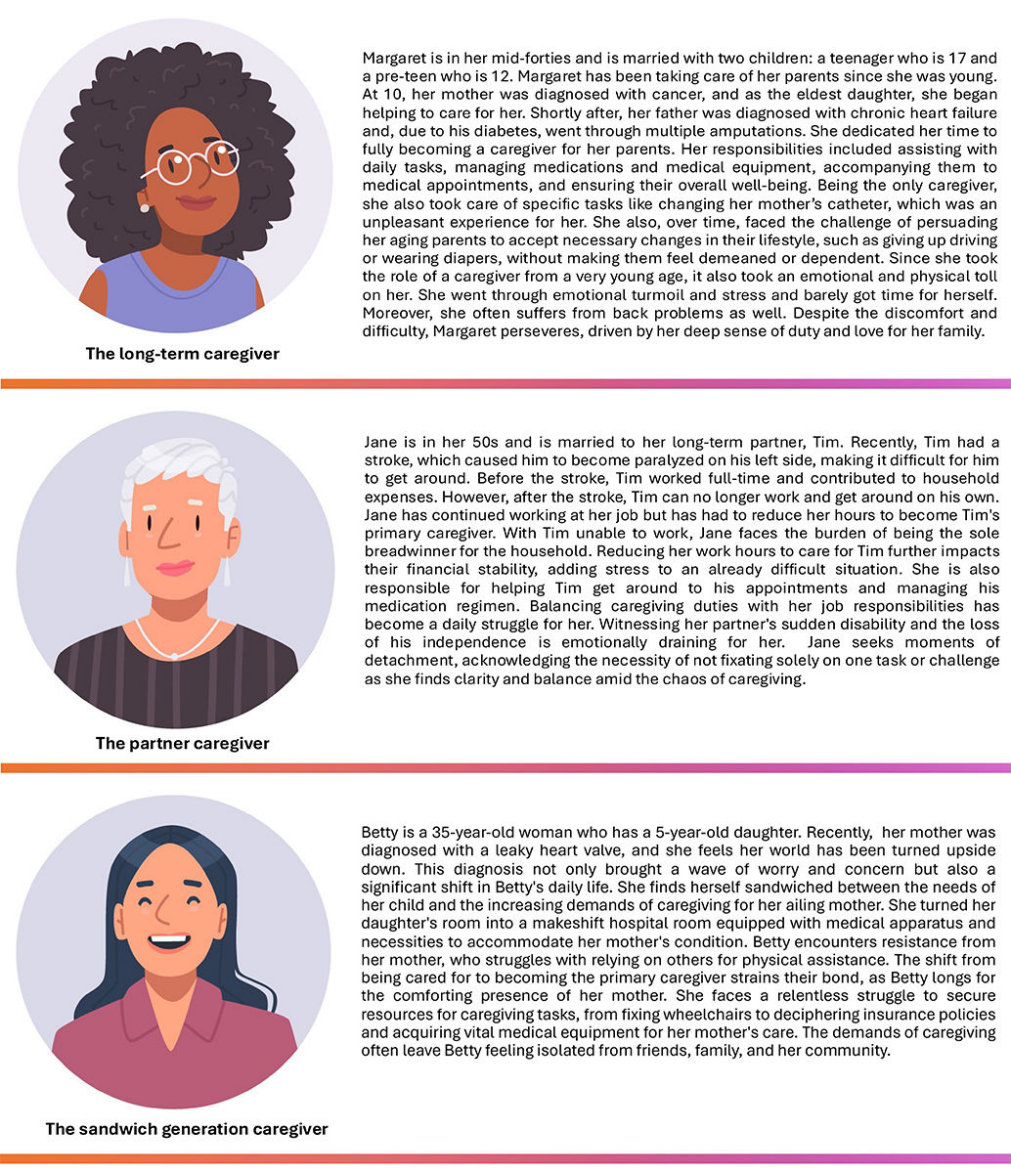
Caregiver personas representing experiences providing care for an older adult friend or family member.

Our participants shared how they came to need care and the emotions surrounding that transition. Caregivers’ experiences included (1) long-term caregivers beginning to care for an ill parent as a child into adulthood and older age, (2) partner caregivers who became primary caregivers of a partner due to a life-altering event, and (3) sandwich generation caregivers who were primary caregivers to both older parents and younger children (Figure 2).

**Figure 3.**
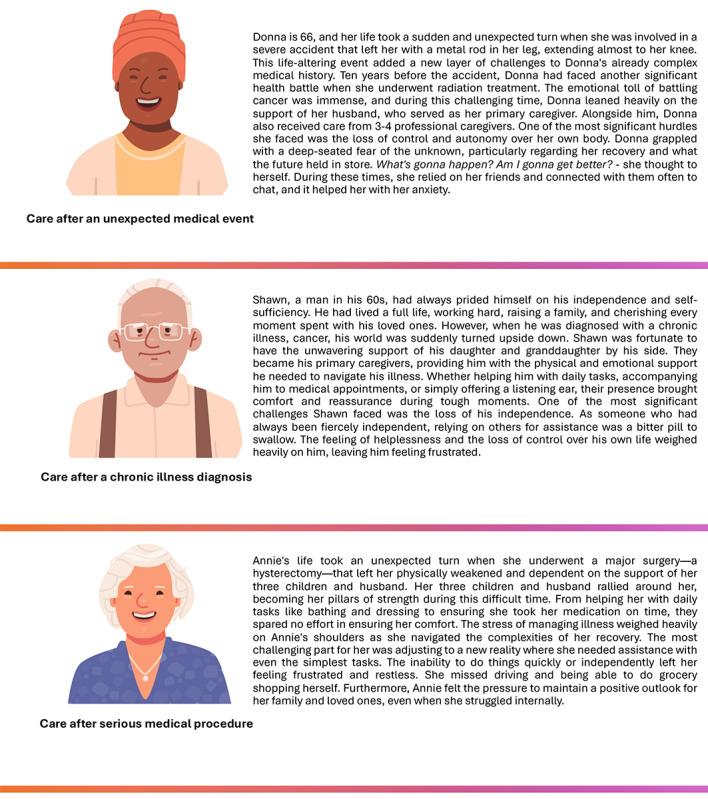
Older adult personas representing experiences collaborating with a friend or family member for informal care.

Care recipients had similar varied experiences ranging from (1) needing care after an unexpected medical event, (2) needing care to support chronic illness management, and (3) needing shorter-term care after a serious medical procedure (Figure 3).

Both older adults and caregivers appreciated the caregiving relationship but noted challenges. For caregivers, most challenges were related to managing and coordinating care, including adjusting to their role, navigating the health care system, and accessing resources (Textbox 1). However, they also shared emotional and social challenges such as feelings of isolation, lack of support, and coping with feelings of being overwhelmed. They also shared that informal caregivers are often not trained; therefore, finding resources and training on different care-related tasks, such as navigating financial policies or communicating sensitive topics, made things more difficult. Textbox 1 lists the range of challenges caregivers listed during the session.

Textbox 1. **Caregivers' caregiving needs and challenges.**

- Lack of resources and information
  - Scarcity of external support networks
  - No access to information about available services
  - Potential loss of income due to caregiving responsibilities
  - Lack of understanding about financial policies
  - Shortcomings of training programs
  - Navigating health care systems and tasks
  - Managing medical appointments and tasks
  - Managing medical equipment and administering medication
  - Convincing older adults to accept help, medication, or medical devices
  - Advocating for the care recipient
- Emotional and psychological toll
  - Feelings of sadness, frustration, and guilt
  - Enduring emotional strain in prolonged caregiving
  - Witnessing decline in health and independence of loved ones
  - Lack of time for personal reflection
  - Dealing with the unpredictability of caregiving tasks
- Isolation and social dynamics
  - Transitioning roles within relationships or role reversal
  - Experiences of social isolation
  - Managing family dynamics
  - Balancing caregiving duties with other commitments
  -  =Lack of self-care
- Communication and sensitive interactions
  - Communicating sensitive topics
  - Difficulty finding solutions to specific issues
  - Convincing older adults to accept help, medication, or medical devices

Older adults shared difficulties they had in transitioning to needing care. They discussed difficulties with losing independence, feeling like a burden, and needing to depend on others (Textbox 2). They shared that the transition to needing care came with a lot of emotional tolls, including adjusting to feelings of social isolation and loneliness, and frequently needing to balance positive and negative feelings that come along with needing care. Textbox 2 lists the range of challenges reported by older adult participants during the session.

Textbox 2. **Older adults' caregiving needs and challenges.**

- Loss of independence and autonomy
  - Loss of independence
  - Dependence on others
  - Sense of loss of control or autonomy
  - Asking for help with basic tasks such as picking up items from the floor
  - Not being able to drive
- Physical and emotional toll
  - Managing the stress of illness or injury
  - Physical limitations
  - Emotional toll
  - Feeling impatient because of not being able to do a task or do it quickly
- Sense of isolation and social disconnection
  - Sense of isolation
  - Fear of future and uncertainty about what lies ahead
  - Feelings of burdening or bothering others for assistance
- Feelings of burden and responsibility
  - Burden of trying to be better for others
  - Feelings of burdening or bothering others for assistance
- Balancing negative and positive emotions
  - Sense of kindness (despite struggles)
  - Sense of kindness (toward those providing help)

During the first session, participants reflected on their caregiving experiences and needs, prioritizing the challenges that VAIs might address. During sessions 2 and 3, caregiver and older adult participants brainstormed different ideas for how VAI assistants might augment and support them as caregivers and care recipients and address the challenges they experienced.

Many initial design ideas from caregivers and care recipients emphasized how they felt VAI could help make caregiving tasks more efficient, or how VAI might help shift caregiver time spent on physical or informational caregiving tasks toward more social and emotional ones. For example, during session 1, participants shared that informal caregivers often lack professional caregiving experience and frequently encounter unfamiliar situations, navigating uncharted territory. Therefore, when asked to prioritize how they felt VAI could address concerns listed from the initial focus group session, caregivers ranked their need for caregiving support as a top priority. They discussed how VAI might help them be more efficient by supporting them with (1) immediate access to relevant information and resources, (2) proactive assistance with caregiving tasks and resources, (3) persuading older adults to accept help, and (4) connecting for socioemotional support as top priorities. A full list of brainstormed ideas is included in Table S3 of Multimedia Appendix 1.

While informal caregivers generated more ideas in their sessions, some overlapped with those of older adults. As with caregivers, older adult participants saw opportunities for VAI to support health information needs with caregiving relationships and to provide socioemotional support for them and their caregivers. Older adults’ ideas primarily focused on VAI providing support through (1) immediate access to relevant information and resources, and (2) connecting for socioemotional support as top priorities. A full list of brainstormed ideas is listed in Table S4 in Multimedia Appendix 1. The following subsections discuss the primary VAI concepts that caregivers and older adults prioritized for supporting informal care.

#### Providing Immediate Access to Information and Resources

Both older adults and caregivers felt that features of VAIs, such as voice input and automation, could help to make health information and other caregiving tasks more efficient. Caregiver participants shared that they manage different types of health information, ranging from supporting the care recipient’s daily health care needs to navigating insurance coverage. For example, one participant gave an example of navigating insurance as a caregiver, C3, W2, “Some things get covered [by insurance]. Some things don’t... You don’t find out until somebody screams [at you] in the financial section of the hospital.*”* They felt VAI could help them find and navigate this and other information more easily and efficiently.

Older adult participants also expressed interest in using existing VAI to support health information needs, such as receiving reminders and alerts for appointments or events, and to provide efficient access to information for managing their health. They recognized the role VAI could play in helping them stay organized and on track with their schedules. They felt that voice-activated information requests, similar to voice-based Google searches, could be convenient for accessing information more quickly and easily. While these initial ideas align somewhat with what current VAI assistants might do, many of the systems on the market are not tailored to support health or care needs.

#### Proactive Assistance With Caregiving Tasks and Resources

Older adult participants tended to discuss ways they foresaw VAIs helping them better manage their health tasks, such as scheduling or quick access to health information. Caregiver participants also envisioned the potential for a more sophisticated VAI information assistant (beyond what currently exists) that could talk to or nudge them proactively, helping them streamline their schedules and manage caregiving tasks. Participants envision this system using their existing health information to intelligently set reminders, manage appointments, and optimize their time, acting as a proactive decision support assistant.

**Figure 4.**
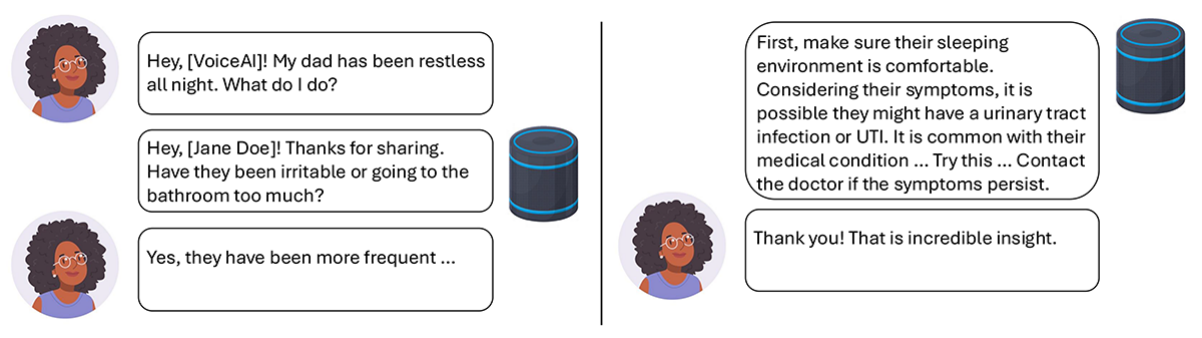
Scenario demonstrating desired interactions between an informal caregiver and VAI that provides advice or tips to help them address caregiving concerns. UTI: urinary tract infection; VAI: vvoice-enabled artificial intelligence.

Caregiver participants discussed the potential of VAIs to reason to anticipate future caregiving duties by efficiently providing relevant information about the older adult patient’s health. One participant described a VAI that could bring together relevant information around a caregiving task to help them complete it. C1, W2 described, “So basically, it’s [the VAI] gonna say like you need a ramp, you need a hospital bed, you need to make sure they have the wheelchair you need. So, it’s gonna give you a list of all the stuff you need and potentially where you get it and who pays for it.” Another participant suggested a VAI that could help troubleshoot problems by understanding patterns and symptoms to identify potential causes (Figure 4). C2, W3 explained, “If it could troubleshoot and identify what their areas of need are, if this person is up all night, what can you do? Are they going to the bathroom too much? Are they irritable? And then suggest: These are the steps that might be able to help them.” They felt this additional support could be valuable in reducing their feelings of being overwhelmed when managing various caregiving tasks and responsibilities.

#### Persuading Older Adults to Accept Help

Caregiving recipients discussed VAI’s potential to support their caregiving routines through virtual companionship; however, their ideas centered on ways virtual companions might leverage human-like characteristics to build trust and persuade older adults they care for to accept assistance, medication, and treatments prescribed for them. One idea presented was using VAI to mimic the voice of a trusted person, such as a celebrity, to encourage acceptance of essential care, such as transitioning to using adult diapers. C1, W2 described, “I’m imagining Harrison Ford making a video saying, Hey, it’s ok to do these things [wear an adult diaper].” Challenging this notion, another caregiver, C3, W2, who also self-identified as an older adult, offered a contrasting perspective, suggesting that “with old age, you get fairly tough skin, so a diaper is a diaper.” They explained that older adults may not find discussing topics such as diapers stigmatizing over time. However, participants generally agreed that leveraging VAI to imitate others to persuade or provide comfort might be helpful in specific scenarios. C1,W3 shared, “...so say the person’s having trouble going to sleep if maybe a book or a scripture passage was read in the caregiver’s voice. I do think something like that in the caregiver’s voice, I think that would be real helpful.”

#### Connecting for Socioemotional Support

##### Opportunities to Support Mental Health and Well-Being

Both older adult and caregiver participants saw opportunities for VAIs to support their mental health and well-being. Caregiver participants tended to focus on how VAIs could support their mental health through reducing caregiving management tasks and through direct mental health support.

##### Supporting Caregiving Management

One set of ideas from caregivers focused on tools that could ease some of the mental burden of managing care. For example, one participant saw promise in the VAI for recording older adult patient requests, ensuring care continuity while reducing the burden of constantly being in “caregiver mode.” Reflecting on their conversations with their mother, one caregiving participant shared that their relationship with their mother shifted over time. They initially had typical mother-daughter interactions; however, because of her dual roles as caregiver and daughter, preserving the natural relationship became more difficult. Therefore, C3,W3 suggested using voice-recorded messages could be helpful, stating “later on it [my role] became more caregiver. And so when we’d wake up in the morning, instead of being like, ‘Hey mom, good morning,’ it would be like, 'I need this, and I need this, and I need that.' So if she could leave a message saying I need all this, then it could open up the morning into being, ‘Hello, good morning. I love you,’ and then I can check the message. So, the request can be via the system so that you still have some of that relationship left.” Participants suggested enhanced VAI systems with more reasoning capabilities could also provide more significant support in caregiving situations. For example, they discussed VAI systems that proactively provide a tailored review of patients’ conditions to first responders in emergencies. C1,W2 described, “If there was an emergency and you called the fire department and they showed up, maybe they’d be able to connect to it [referring to the VAI], and that would immediately tell them what the patient’s conditions are, what medications they’re on.”

##### Supporting Caregiver’s Mental Well-Being

Another set of ideas revolved around how VAI might directly support caregivers’ mental health needs. Caregivers explained that being in a caregiving environment often comes with much stress, and therefore, they saw opportunities for VAI to support self-care and mental wellness. Participants suggested ideas for VAI to help them curate self-care plans, with occasional reminders to care for themselves or to complete self-care activities. C3, W2 shared, “It [the VAI] could give me a reminder to go do something for myself as self-care and maybe not for long periods of time because as a caregiver you don’t have a lot of time ... also like have a self-care plan so that while it is reassuring, it can also walk you through yours.” C3,W3 suggested using VAI as an emotional support, “I want this thing [VAI system] to comfort me in some way to. I want it to be like, look, I’m here to help you take a deep breath. It’s gonna be OK.” As shown in Figure S3 in Multimedia Appendix 1, participants brainstormed ways VAI systems that identify stress cues could offer positive affirmations or prompt caregivers to take breaks when needed. For example, the VAI could also converse with them to comfort them in difficult times. In addition, they explained that caregivers are often isolated, and the VAI could help connect them to support groups or people in similar situations.

##### Supporting Older Adult Self-Care

**Figure 5.**
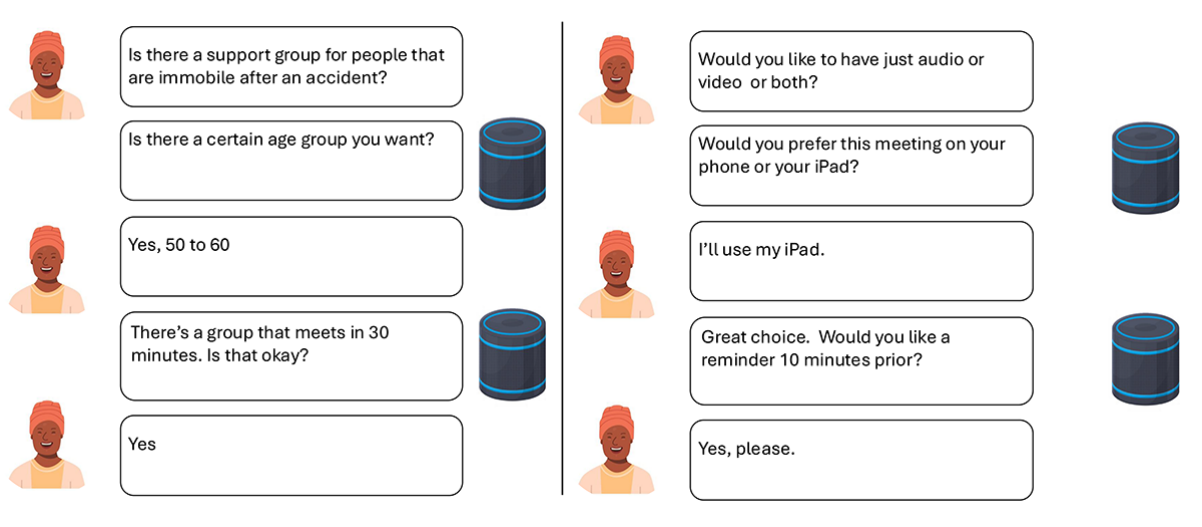
Scenario representing a conversation between a person and a VAI assistant, discussing joining a support group for older adults experiencing mobility changes. VAI: voice-enabled artificial intelligence.

As with caregivers, older adults also felt VAI could help support self-care and well-being. They shared ideas for VAI systems to help them find leisure activities, viewing them as an accessible resource for quickly finding books, movies, or other media to enjoy as part of their well-being and self-care. Older adult participants, as with caregivers, also discussed generally using VAI for emergencies to establish instant connections to emergency services or loved ones, especially in times of crisis, when they expressed the need for an instant connection to emergency contacts. However, as with caregivers, most ideas centered around VAI as a facilitator for connecting with others (Figure 5). They shared ideas about the possibility of VAI helping them connect to their friends or loved ones through voice commands on a screen. O2,W2 explained, “I think [VAI] could be helpful in the emotional sense. If you're in bed and you don't wanna get up, you could always say call John.” O3,W2 made similar suggestions, “It would be uh trained to be supportive of all their emotional needs and [when] you kind of shut down . she[the VAI] . on how much can we help this person who seems to have bouts of crying or they’re really depressed and get to know them and see what the problems are and then [be] smart enough to try .and act out what would help that person get back to a stable emotional point.” Participants also discussed the potential for VAI to connect them with people who have experienced similar situations automatically. O2,W2 later asked, “Is there a way that with AI, you could say connect me to the support group for people with recent car accidents, for instance.”

##### Supporting Older Adults Through Virtual Companionship

Older adult participants envisioned new ways in which VAI could enrich their lives as care recipients by providing companionship and social support. Participants envisioned new ways to engage in dialogue and interact with VAI as a virtual companion, which they felt could help alleviate feelings of loneliness during periods of solitude or immobility. Participants described a VAI system as an “AI girlfriend” (eg, a romance chatbot or robot). O3,W2 explained, “...they say [AI girlfriends] actually learn what they[the human partner] need and are looking for, and they’re able to change how they respond to meet those needs. And if they can do that with AI girlfriends, I’m guessing they could absolutely do it with people that were depressed or down emotionally and actually learn what it is that that person is going through.*”* However, participants also expressed apprehension about the possibility of humans developing emotional attachments to AI entities. O1,W2 posed, “The question is, can AI really meet someone’s emotional needs?” At the same time, they expressed discomfort at conversing with a system rather than a human, stating, “Some of it [VAI] also scares me when they pretend, they’re human or sound human.”

Participants were also unsure whether a virtual companion should be embodied. One participant suggested embedding a VAI in robotic entities, such as robotic puppies equipped with AI to interact verbally and physically with individuals and to simulate lifelike behavior, such as mouth movements or eye blinking. In response, another participant shared that they preferred a discreet, compact device rather than a visually conspicuous one. They voiced concerns about potential discomfort associated with being constantly monitored by a visible robotic assistant, highlighting worries about their appearance or attire. 02,W2 mentioned, “I’m kind of stuck on something like a small device .I wouldn’t necessarily want to see me . that’d be another thing to think about. Am I dressed or whatever? And I like to think that even though Alexa hears everything, she can’t see anything.” Older adult participants also considered that, while virtual assistants such as Alexa may capture audio, they lack visual capabilities. The idea of a small voice assistant device appealed to them because it is nonintrusive and occupies minimal space in a room.

### VAI Can Help, but it Is Not Enough

Older adult and caregiver participants viewed VAI as a potentially valuable tool to support their caregiving activities. However, participants also consistently pointed out that VAI would not be comprehensive enough to support the complex environment of caring for an older adult. Therefore, both groups also noted their desire for VAI to be considered broadly as one of several tools that might seamlessly integrate into a more extensive system. We learned that participants’ reasoning for this perspective was related to several factors, including a desire to support various access and accessibility needs, to promote more integrated care, and to ensure fair access.

**Figure 6.**
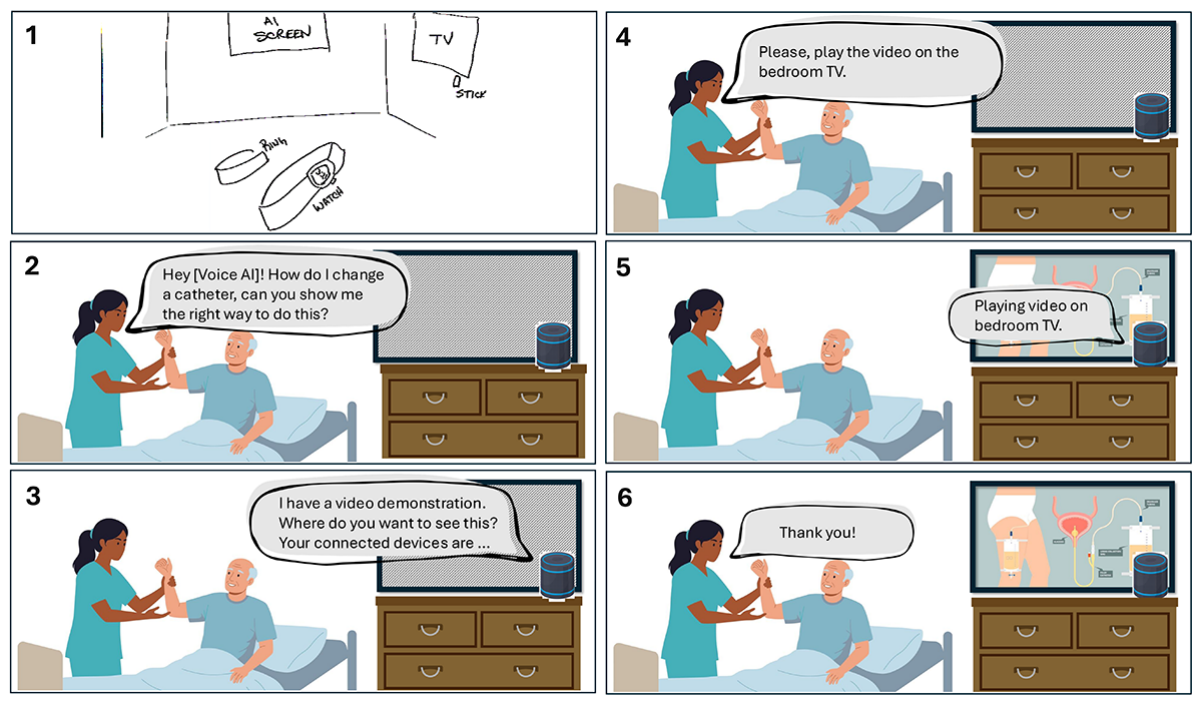
Scenario representing a VAI caregiving ecosystem that connects other devices, such as smartwatches, televisions, and voice assistants, to coordinate various care tasks. On the wall, there is an “AI SCREEN” and a “TV” with an Amazon Fire TV Stick labeled “STICK.” Next to it are a “RING” and a “WATCH” displaying the time. Followed is a storyboard of the VAI ecosystem scenarios participants discussed, showing a person interacting with a VAI to play a video demonstration of how to insert a catheter. AI: artificial intelligence; TV: television; VAI: voice-enabled artificial intelligence.

Participants suggested that VAI could be used as a tool to help them connect with multiple screens, noting that it may also be important for some users to have visual aids to enhance assistance and support varied accessibility needs. Additionally, participants noted that they sometimes preferred video demonstrations for tasks that might benefit from seeing them performed (eg, changing a catheter). They suggested that VAI-enhanced visual guidance might help them better perform the caregiving tasks (Figure 6). C3,W2 explained, “I would prefer having someone with a video showing me [for some tasks] every time [I was changing a catheter]. I like to see how things are done.” These examples suggested that some participants preferred prioritizing the modality of information delivery that best supports the care task at hand, rather than defaulting to VAI alone.

Caregivers also expressed the need for VAI to connect to multiple devices, enabling a more comprehensive approach to care support. C1,W3 discussed that it would be essential for the solution to have “different modalities because if something is also tracking their vital signs and everything, it has to be attached to the patient as well.” They asked their group, “So what does that look like?” C2,W3 noted, “I personally would say again, things are gonna get smaller all the time, but like a ring, … or, you know, a watch. The necklace [fall necklace] got taken off much of the time. It got yanked. An Apple Watch?” C3, W3 explained, “The connectivity[has to be] built in with at least the phone.” C1, W3 summarized, “Maybe this is like one program that can get attached to different equipment. Like your ring or watch or your TV, for that matter. And it can also attach to, like, you know, how Alexa can control your lights and everything. So, like something that can control different things differently, because, like it, when I was taking care of my mom, um, I’d have her watching TV, so I would want two separate units so that she could be watching TV while I was maybe watching something like a procedure.” The participants drew a sketch illustrating their vision of a device ecosystem they would prefer to see integrated with VAI (Figure 6).

In addition to caregivers’ perspectives, older adults emphasized the importance of VAI seamlessly integrating with the devices and technology they already use in their daily lives. For example, older adults wanted to give voice commands to the AI system to find information or pull up a book to read on their iPad (Apple Inc). They explained that the difference between their envisioned and existing VAI systems is that the primary agent assists in managing and connecting these different systems. They felt that integrating VAI could extend the flexibility and convenience of other devices, making care and wellness tasks easier for them overall. O2,W2 noted, “Maybe something that you can turn on if you want to talk, that could turn on through your television or through an iPad. So that if you want it to pull up something for you to read, for instance, Alexa, find me a good book on whatever. I mean, that’s going pretty far out.” In describing an interaction with the system, a participant demonstrated how they envision the VAI providing options when choosing a mode of interaction, for example, connecting them to a support group. Overall, participants view VAI as a tool within a broader ecosystem that supports care environments, and therefore proposed that a multimodal approach integrating VAI with other technologies would be ideal for addressing the complexities of caregiving support.

### Exploring Ethical Values and Dimensions: Ethical Critiques of Envisioned VAI Concepts

#### Balancing Needs for Informal Care Support and Ethical Concerns

In the final workshop, participants provided ethical critiques of their VAI concepts, reflecting on and discussing potential ethical concerns about integrating solutions they designed into informal care. During workshops 2 and 3, we observed participants navigating various design tensions as they brainstormed ideas, revealing some contrasting perspectives and conflicting opinions regarding how VAI might support the care relationship (or not). For example, when suggesting ideas for VAI as emotional support, we found that participants questioned whether VAI should provide emotional support.

Further, while both older adult and caregiver participants remained positive about VAI’s potential efficiency, in workshops 2‐3, we saw some different ideas of how VAIs might support their care. We expected some differences due to their experiences in their caregiving roles, although many of our caregivers were also older adults and provided perspectives from both roles. However, we found that when discussing ethical values, older adults and caregiver participants shared overlapping ethical concerns and expectations about how VAIs might handle health data and system transparency, as well as varied older adult needs, system trust and accountability, privacy and confidentiality, fairness and affordability, and shared autonomy. Therefore, participants highlighted a need to balance efficiency and ethics in designing and implementing such technologies. They discussed that if systems could address their ethical values, they would consider integrating VAI into their caregiving routines in the future.

To structure the discussion on ethical values, we report the ethical values and dimensions participants discussed to align with the 11 dimensions of AI ethics by Jobin et al [14], which include transparency, justice and fairness, nonmaleficence, responsibility, privacy, beneficence, freedom and autonomy, trust, sustainability, dignity, and solidarity. Similar to Jobin et al [14], we found that many of these dimensions overlap; however, of the eleven dimensions Jobin et al found in literature, our participants discussed seven dimensions: (1) transparency, (2) privacy, and (3) trust (including (4) nonmaleficence and (5) responsibility), (6) justice and fairness, and (7) freedom and autonomy.

#### Transparency, Privacy, and Trust

##### Overview

We found that participants considered the ethical dimensions of transparency, privacy, and trust to be essential yet complementary components in designing ethical VAI systems for informal care. While participants often discussed these components separately, the main takeaway was that the systems needed to provide avenues to build trust among users through transparent interactions, respect for privacy, and accurate information. Participants voiced a desire for clear, honest communication from VAI systems handling health data to ensure transparency about the systems and actions that help them better trust the system. At the same time, participants expected that systems would build trust by respecting their privacy, both by ensuring data confidentiality and by promoting privacy best practices. Relatedly, participants expected that VAI systems should proactively cultivate trust by ensuring the accuracy of information through personalization and contextually informed responses while limiting misinformation.

##### Ensure Sufficient Transparency of Health Data and System Actions

Discussions around transparency values related to data handling practices and knowledge of “how the system worked.” Participants felt it was important to understand how the system handled their data, especially any health-related data or activity, including voice recordings of the surrounding environment or video capture, as they may contain sensitive data. Participants also wanted information about sources of responses and information from a VAI system. In a discussion about transparent communication of sources, a caregiver, C1,W4, stated that “I think there will be a lot of users that would not even care [about a VAI answer source], but there are people that would be like me, I really want to know where they got this information.” However, while participants felt that knowing the source would be necessary, there was no consensus on how the system should provide it. The participant later noted that they would not want source information to be intrusive or prominently displayed, stating, “I think you want access to it, but don’t put it in my face.” Another participant, C2,W4, acknowledged that in critical or sensitive situations, the immediate need for assistance would likely take precedence over reviewing sources, stating, “I would absolutely wanna research so much of this information, but like in a situation that you’re just thrown into, you just want the help, and you’ll research it a little bit later.”

Older adult participants shared that they often lacked understanding of the workings of AI systems and expressed that it would be important to have broader support for understanding the technology’s capabilities and limitations. They emphasized the need to be informed about what they can and cannot do with it, noting that this has been an issue with past VAI technologies. O1, W4 referenced an incident in which Alexa altered its voice during the interaction, prompting them to question whether it was mimicking or mocking them. They shared, “this was just an experiment with my Echo, I just said, I love you. And she came back in a human-sounding voice. I thought that was kind of interesting in a weird way. Was it mocking?” Therefore, in addition to being transparent about answers and recommendations, participants also valued having a general understanding of “How an AI works.” The participant mentioned, “What would make me trust it [VAI] is education, knowing more about it, reading more about it, learning how they do it.”

##### Mitigate Privacy and Confidentiality Concerns Around Sensitive Data Handling

Caregivers and older adults voiced concerns about the substantial volume of sensitive data that VAI technology would need to effectively assist them with some caregiving tasks. They believed that accessing that volume of sensitive data carries the inherent risk of data breaches associated with storing and processing such confidential information. C2,W4 participant noted, “We’re asking it to be kind of the keeper of all information. What if it were hacked?” Additionally, caregivers proposed implementing an authorization process for VAI technology, given its handling of health care information, noting that this process would ensure that only authorized individuals can issue commands and receive information. C1,W4 explained, “...if a new person is there [attempting to access personal health data], they should be authorized in some way to use this technology... the technology should only interact with the people it has registered as, like the usual voices.” Here, the participant suggested that voice could be used as a unique identifier, similar to fingerprints or face recognition, to restrict or grant access to people using the system.

In one of the discussions, older adults contemplated whether asking sensitive questions or seeking personal information (eg, asking about “finding an economical nursing home”) might pose additional risk compared to a traditional online search. They explained that they would be more reluctant to disclose certain types of sensitive health information to AI systems. O3,W3 noted, “I don’t think I’d ever give my charge card numbers to AI. I guess one of the privacy things I thought about was just simply, do I want AI to know?” They indicated a reluctance to entrust AI with sensitive information, such as credit card numbers used to pay medical bills, even if it would be convenient, preferring not to “throw it out into the World Wide Web.” They acknowledged that while some companies claim not to share information, they are often skeptical of such claims.

##### Cultivate Trust in the System and Be Accountable to Recommendations

Participants mainly discussed trust and accountability values through the lens of accuracy, highlighting their importance. Participants felt that, in a health care context, a VAI would need to be as accurate as possible. They felt this was true for any health care technology but discussed the unique benefits and challenges of accuracy in a VAI system.

Older adult participants discussed their worries about receiving incorrect information from a VAI system, especially for sensitive topics such as health. Caregivers and older adults, therefore, shared apprehensions about reliance on VAI technologies in caregiving situations. O2,W4 stated, “If I got a response that was totally crazy... I wouldn't use it anymore,” explaining that if the system provided an answer that they felt was inaccurate, it would impact their use. O3,W4 discussed that in the news, they heard anecdotes about VAIs that might fabricate responses to appease the user. One older adult participant shared, “the little bits that I’ve really read about AI is that it tries real hard, and if it can’t find what the legitimate answers are, it’ll make stuff up to try and make sure that it satisfies.”

Some participants also discussed the potential value of having a system that could personalize responses. They emphasized that they would value and trust personalized VAI systems that could provide contextually relevant responses tailored to individual needs and situations, rather than more abstract, generalized responses. A C1,W4 noted, “just having the technology to be able to individualize it to the environment, the person, and... their situation, I think. that’d be pretty accurate.” In care-related contexts, participants felt that a degree of personalization was essential for providing more relevant responses to aid decision-making.

Older adult participants expressed concerns about the lack of fine-grained control they feel creators have over how AI might respond to queries, leading to inaccurate and unexpected responses. O3,W3 shared, “...I think AI, right now, I’m afraid they [big companies/AI developers] don’t know how to control it.” Similarly, they explained that they knew humans ultimately created AI systems, but felt humans could be limited in their ability to control how AI evolves because of how AI systems learn. O3,W3 said, “I know there’s a programmer who writes the programs, but the way AI is evolving, it’s really no longer under the control of a programmer; it’s evolving on its own.” As a result, participants mentioned that they would still consider AI-generated answers to some extent but would approach them with skepticism and exercise caution. Moreover, C3,W4 mentioned that they felt a trusted medical organization, such as the American Medical Association (ie, a trusted nonprofit organization in the United States consisting of medical professionals, students, and other stakeholders focused on issues related to patient care and policy), should validate and endorse any VAI caregiving system.

### Justice and Fairness

Participants noted that they would expect ethically designed VAIs to consider the diversity of experiences in the design, including accessibility needs and inclusive design for older adults. While affordability was not identified as a component of current AI ethical dimensions in prior work [14], participants also noted that cost should be taken into account, as it could further widen gaps in access to health care resources.

#### Be Inclusive of Varying Older Adult Needs

Regarding accessibility, participants felt that while a VAI system could improve accessibility in some cases, it could also create additional accessibility challenges in others. A participant in the older adult session highlighted the potential benefits of voice commands in technology, noting that they may experience vision limitations as they age. O1,W4 explained, “...someday I won’t be able to see very well, it enters my mind, and I would need help. ...It’ll be helpful for you in a way if you can operate something with your voice, let’s say you can just say, hey Alexa, connect me to this friend of mine, and they just do it.” However, participants felt that if the VAI included the smaller screens found in commercial devices, access challenges could arise for people with visual disabilities. While they noted that the VAI could leverage voice as an alternative input method, larger screens could facilitate easier navigation and interaction and open possibilities for new functionalities, such as virtual doctor visits. O1,W3 explained, “So if I was developing a system and instead of Alexa, it was called nurse K or whatever that. Ok, that system and I think they’re gonna move to screens on pretty much everything.” O3,W3 agreed, noting access to a screen can be helpful, “Uh, I think Ring [the camera system] is one that comes with screens now. And so, it does the same thing Alexa does, it can search the internet or play songs or whatever, but it also has video and .I think you can get it on your phone or your laptop or iPad.” Participants also emphasized the importance of adequate volume levels to address hearing loss among older adults. Therefore, participants raised concerns about balancing the introduction of a new interaction modality, VAI, with its inherent access challenges.

Participants also discussed values related to the diversity of speech when interacting with a VAI specifically designed to assist with care. Caregiver participants stressed the importance of representing diverse dialects to ensure the VAI system sounds natural and is easily understood, as overly mechanical voices can present challenges for users and make them less comfortable. C1,W3 explained, “because a lot of elderly people have hearing loss. So, I think it’s important that we make sure the volume is good for them . and that the person’s dialect is represented. That’s critical because if it sounds not too foreign but too mechanical, that could be a challenge too.”

#### Make It Affordable, Promote Fair Access to Health Resources

Participants felt that affordability was an ethical concern for VAI systems that support caregiving. Participants felt that while the VAI system had the potential to improve efficiency, it would also incur higher costs. At the same time, cost is not always a factor formally considered in discussions of ethics. Participants noted that affordability could be a factor in determining who has access to these systems. Therefore, they related cost and affordability to justice and fairness in this context. They felt it was important that, if these VAI systems became available for caregiving purposes, they should be accessible to all who might elect to use them, so that all who wish to benefit might do so. Therefore, caregivers particularly emphasized a preference for integrating health-related VAI systems with insurance or existing health care systems, such as “MyChart,” a patient-centered health record and messaging system, which already centralizes patients’ health care information:

*I go to Saint Francis, and they have something called MyChart … could it tie into something that’s already existing like that …like a booster to that system. It would be good if it could tag on to what already exists out there that people are familiar with and be an enhancement. That would give you some legitimacy*.

[C2,W4]

Moreover, older adults expressed apprehension about the future maintenance costs of such systems, especially because they felt their vigilance about maintaining subscriptions might decrease with age. A participant in the older adults’ group, O2, W3, gave an example of how they were surprised to find that Alexa could facilitate games such as Jeopardy; however, they were surprised when it prompted them to start a free trial that later required a monthly payment of US $7.95. They shared, “I don’t know much, and I asked my echo what she can do and, you know, it’s funny. I said she could do games, and so she and I picked Jeopardy, and the music played, and I thought, well, this is really neat for people who can’t get up and around, and it said you can start a free trial for a week. And then after that, it’s $7.95 a month. Never occurred to me that there would be extra things on there that would cost money. I was kind of surprised by that. Another observation is dependency.*”* Participants noted that in situations where they might rely on these types of VAI devices to support their care, they sometimes face a dilemma between continued care support (ie, from the system) and managing ongoing costs, which may limit access.

### Freedom and Autonomy

Participants’ discussions of freedom and autonomy mainly focused on the right to choose where and how VAIs are integrated into the care relationship, and on having sufficient autonomy to prioritize human relationships over functional support.

Participants voiced concerns about balancing technology-enabled caregiving assistance and the human touch. While acknowledging the potential benefits of technological support in caregiving, caregivers emphasized that it was essential to ensure that any technology, including VAI, does not overshadow or replace human interaction and compassion. They noted that the system must respect their values and rights to care autonomy (eg, rights to self-govern their care environment and experiences). O1,W2 shared, “I think the human touch and kindness is very important.” C1,W4 shared, “It would be really important the individual is interacting with it [the VAI] instead of it taking over because it’s real easy to do that.”

Caregivers also emphasized that if technology aids caregiving activities, it should not be overly assertive, especially when empathy, compassion, and understanding of the situation and context might be necessary rather than a strict ultimatum. They gave the example of a VAI that might recommend an individual’s water intake based on a predefined number of ounces, using established health guidelines. C1,W4 shared that it may not be productive for the VAI to recommend, “The person needs to drink a gallon of water a day when you’re lucky to get eight ounces of water in them. I guess you wouldn’t want it to be too aggressive.” They felt that this was a situation where a human would understand the situation more holistically, and that an assertive AI might end up being discouraging rather than motivating.

Older adults expressed appreciation for the assistance and accountability that VAI technology could provide, including reminders to engage in beneficial activities such as stretching. However, they discussed that they felt it was important to maintain control over the technology’s level of involvement in their care and have the option to override its actions if they feel it becomes too intrusive or overpowering. O3,W4 shared, “If it goes too far and I’m unhappy with it, I just want that kill switch to say no, you’re done.”

## Discussion

### Summary of Findings

In this study, we used a 3-phase research approach to explore the potential of VAI for informal caregiving and identify ethical dimensions that might impact adoption. We aimed to answer the following research questions: (RQ1) How do participants perceive the role of VAIs in supporting caregiving tasks? (RQ2) What values do caregivers and older adults have around VAI assistants that support day-to-day care tasks? (RQ3) What ethical concerns do caregivers and older adults have about VAI assistants that support day-to-day care tasks?

The findings illuminate a core tension between the perceived benefits and efficiency of VAI for supporting informal care and the ethical considerations surrounding its implementation. Participants identified several ways VAI could enhance caregiving efficiency, streamline caregiving tasks, and provide emotional, social, and training or educational support. Nevertheless, caregivers and older adults express apprehensions regarding the ethical implications of AI-driven caregiving solutions. Based on these findings, we also provide design considerations regarding the role of VAIs in assisting informal aging care and in addressing ethical concerns from a harm-reduction perspective.

### Role of Voice-Activated Assistance in Informal Care: How Can Voice-Activated Systems Help?

Related to RQ1 and RQ2, we found that the primary appeal of VAI systems was the potential capacity to streamline caregiving routines, allowing caregivers and older adults to manage care tasks quickly and conveniently. Participants felt that this efficiency is particularly valuable when timely support and task management are critical. This finding aligns with other studies in the health care context, in which researchers have found that older adults desire VAI features that help with caregiving tasks, such as scheduling and appointment management, or medication reminders, for perceived convenience and efficiency [4,8,10,13]. We also found that VAI was viewed not only as a practical tool but also as a potential source of companionship to mitigate the feelings of loneliness and social isolation prevalent among caregivers and care recipients. Although not explicitly situated in the context of informal caregiving, prior work has explored the use of VAI as companions for older adults [4,5,50-53] and its potential to support social connections [54,55]. Rudnik et al [9] explored how a VAI system might benefit care conversations between older adults and caregivers. Yet our work extends prior work by finding that designing VAI for companionship and supporting social dimensions within a caregiving relationship will likely require careful additional exploration of existing relational values that form the foundation of the caregiving relationship. For example, participants envisioned VAI as a way to support socioemotional aspects of informal care, but also raised concerns about privacy, emotional attachment, and the erosion of human connection that VAI might bring. Therefore, our work suggests that, when designing, there needs to be a broader ethical discourse on integrating VAI and on the broader user values surrounding social relationships and human connectedness in caregiving contexts. Future work is likely needed to better understand those values and how they might shape the design of VAI technologies for informal care.

While some work has explored how VAI might be integrated to reduce caregiver burden [9,13], we also found that caregivers felt that a VAI system could help address the emotional toll of providing informal care. They saw opportunities for VAI to be a proactive companion in helping them remember to practice self-care and find related opportunities. Examples included proactively conducting self-care checks or guiding them through exercises when they felt overwhelmed. Additionally, we found that part of the emotional toll of caregiving lies in learning to provide care, as one must learn on the “job.” VAI was also seen as potentially useful for helping caregivers adjust by providing an efficient way to obtain the training, resources, and information they need to function as informal caregivers. For example, VAIs might be integrated within a do-it-yourself smart home setup to provide caregivers with a hands-free way to interact with educational materials and resources. Researchers have found that older adults see potential in conversational agents to support access to personal health records and to help find and understand health data [27]. Voice-enabled systems might integrate with patient portals to create more accessible interactions with the health data included there. Future work should explore more ways VAI might assist with education, training, and support in informal care.

Our research findings also suggest that participants generally felt that relying solely on the voice modality is insufficient for fully addressing the complexities of a caregiving environment. While they felt that VAI provides a valuable interface for interaction, they felt its effectiveness would likely be enhanced if integrated into a broader care ecosystem that included multimodal features, such as visual displays, tactile feedback, and contextual cues. They discussed how often different caregiving tasks require different approaches and that different environments might have different needs. Therefore, in the future, it may be beneficial for designers to consider ways VAI might act as a facilitator of care tasks within informal care environments, for example, by exploring interoperable or multiplatform facilitation and connecting disparate devices into a connected ecosystem. For example, participants suggested that voice input and output be a way for them to seamlessly connect with different devices that support their self-care, health, and well-being, including wearable devices such as smartwatches and smart rings, instructional videos for caregivers, and smart televisions, to allow older adults and caregivers a more comprehensive picture of their health, while also supporting older adults’ independence and reducing caregiver tasks.

### Assist Me, but Do No Harm: What Ethical Values Should VAIs for Informal Care Consider?

Users’ ethical concerns about AI technologies are not new and have been raised in other research [14]. For example, within health care, researchers have explored ethical concerns around using conversational agents for health-related areas such as mental health [56] and more broadly care providers [57] and public health [58], noting potential risks such as algorithmic bias, user harms, and privacy concerns due to users’ unawareness that they are interacting with a virtual agent. Others have noted safety concerns, particularly in voice-only applications, due to a lack of transparency [36]. Privacy remains a core ethical concern among those using VAI systems, along with others related to personification and emotional connections to machines [19]. In parallel, general concerns about user personification of VAI technologies are well-known, with the HCI literature suggesting that the “human-like” aspect of voice user interfaces can sometimes be an enhancement or a detriment to ethical design, depending on context [59]. Therefore, while VAIs can have promising applications in health care, it is often not well known how ethical concerns vary among users and across contexts [19]. This has been found especially true within health-related contexts, where users tend to hold AI technologies to different standards depending on the context, given the sensitive data passed and the potential harms (eg, safety-related) that might arise from its use [33].

Related to RQ3, our findings highlight that VAIs are beneficial primarily because of perceptions of improved efficiency and social or emotional support for informal caregiving tasks. However, our research reveals a critical tension between the efficiency gains offered by VAI in caregiving and the ethical considerations that accompany its use. Therefore, the efficiency provided by VAI must be balanced against these ethical considerations, ensuring that the technology remains a tool for empowerment rather than a source of control or intrusion.

Within health care, the idea of interventional harm is typically associated with safety or injury [60]. However, in recent years, researchers have noted the significant potential harm of social-behavioral interventions that may not have immediate safety concerns, but due to the disruption they cause to complex social systems and needs for human agency, can lead to unintended effects [60]. Therefore, more emphasis has been placed on unearthing non–safety-related and unintentional harms to explore the potential impact of those concerns on intervention success (eg, acceptance and adoption) [60,61]. For example, Allen-Scott et al [61] found that for public health interventions, five high-level unintended harms are often associated with unsuccessful public health intervention deployment, including (1) physical, (2) psychosocial, (3) economic, (4) cultural, and (5) environmental which often occur for reasons including lack of community engagement when developing the intervention and ignoring root causes which lead to a lack of understanding of what users want. Examining harm is also a topic that has been explored among VAI technologies more generally [62,63]. For example, Reeves [62] examined the trade-off between framing VAI interactions as conversations and the potential for user harm. Aligned with these efforts, we summarize the ethical values highlighted by older adults and caregivers into broad categories of potential unintended harms that may ultimately lead to nonacceptance and nonuse of VAIs for informal care.

### Physical Harms

Aligned with prior work on VAI systems, our findings suggest that assessing the potential safety-related harms of health-related VAI technologies remains paramount to users. Safety concerns associated with VAI use in health have been raised in studies where physicians note the potential harms to patients who rely on voice assistants for health information search [36]. However, beyond safety implications associated with receiving inaccurate health information or recommendations, our participants noted broader transparency issues that should be considered, such as helping users understand how decisions are made and/or the data privacy consequences that could affect their ability to receive care and resources (eg, breaches that impact insurance availability).

### Financial Harms

Two categories related to finances emerged from our data: (1) the potential harm associated with data privacy breaches of sensitive information, and (2) increasing inequities in access to health resources due to limited financial resources to adopt health VAIs. Therefore, the implications of a financial data breach involving VAIs that could affect a user’s financial wellness should be considered. Additionally, while not often listed as an ethical consideration for AI generally [14], others have found that cost is an aspect users consider in VAI adoption [11]. Therefore, cost and other financial factors should be considered in efforts to reduce inequities in access to caregiving resources. Mitigating the trade-offs between the efficiency gains and the ongoing costs of adoption would be essential to ensure that the benefits of VAI reach a wider population of users.

### Relational or Social Harms

Our findings align with the informal caregiving literature, which notes that the social, relational, and emotional aspects of informal caregiving are just as important as physical caregiving tasks. However, we found that human autonomy concerns [64], such as shared governance of caregiving tasks, emerged when discussing the social or relational elements of VAI design for informal care, including those related to physical tasks that required social interaction. These findings suggest that users may value VAI in ways that can support social and relational engagement if it is designed in a way that does not overstep the boundaries of what makes certain aspects of an informal caregiving relationship a uniquely human endeavor (eg, human touch, social connectedness, human relationship building, and inclusiveness). Other researchers have found that the social aspects of VAI technologies, such as how they engage users, can impact perceptions, for example, among marginalized groups [65]. Therefore, while providing human-like characteristics to VAI systems is generally thought to have potential advantages for supporting health-related applications [66], our findings suggest that it may be necessary to further explore the impact of adding human-like characteristics on users’ perceptions of autonomy when governing their caregiving relationships.

### Design Considerations for Ethical VAI for Aging and Informal Care

Based on participants’ suggestions, to further address RQ3, we also provide design implications for addressing their desired interactions and ethical values related to (1) transparency, privacy, and trust (including nonmaleficence and responsibility), (2) justice and fairness, and (3) freedom and autonomy when engaging with VAI, which supports informal caregiving practices.

We suggest that designers and developers encourage trust by incorporating features that help users assess the credibility of the information they receive (D1). Prior work has found that limited access to source information makes it difficult to assess the credibility of VAI systems for health [28,67], and the ability to view source information can reduce those concerns [27]. As VAIs - often inherently have less transparency due to the lack of visual guidance, VAIs designed for informal care should consider ways of integrating transparency within conversational design, such as allowing users to ask questions verbally about how their data is used or how the system works, or through multimodal approaches that allow users to better understand who has access to their data or fact-check information sources.

Older adults’ privacy concerns related to VAI technologies have been well-documented in the literature [68,69]. Our findings suggest that older adults and caregivers have similar concerns about VAIs that are integrated into their care processes, including unauthorized access to personal health data. We suggest that designers and developers consider the dynamic privacy needs of potential users of these systems, including older adults, informal caregivers, and others involved in the informal care process (D2). Due to the sensitive nature of health data, VAI systems should implement industry-standard security protocols and help users build greater trust in the system through transparent, clear communication about privacy policies. Systems should also emphasize user autonomy in data sharing, allowing users to control how their data is shared with others and third parties. Systems can also leverage VAI biometrics to prevent unauthorized access to sensitive information.

Prior literature also notes that older adults desire personalized experiences with intelligent voice technologies to support efficient task completion [11,21,22]. For example, Brewer et al [22] found in their study of older adults’ use of current voice assistants for health information seeking that older adults asked for more personalized health information, feedback, and recommendations. In clinical settings, extending voice capabilities to personalize experiences has been found beneficial [70]. Our findings suggest that designers and developers should consider leveraging personalization to address diverse caregiving needs (D3). For example, VAIs might leverage contextual cues from users and environmental information to provide more personalized responses (eg, gauging mood to support caregiver wellness or older adult well-being).

Finally, balancing assistance and control is a longstanding open challenge in systems that automate tasks [71], leading to approaches such as mixed-initiative interaction [72] and, more recently, a focus on understanding human autonomy in AI systems [64]. Studying autonomy in AI-enabled systems has also been longstanding in technology designed for older adults [73-77], given frequent discussions of independence and agency as well as balancing AI and the human aspects of care [78]. Findings suggest that the successful integration of VAIs into informal caregiving relationships requires researchers to consider broader ethical values around the importance of the human aspects of caregiving relationships (D4). Further, participants noted that VAI alone is not enough to support the complex environment of informal care. Instead of viewing VAI as a standalone solution, designers should explore ways to leverage its capabilities to support more holistic care within the caregiving environment (D5). This can include exploring ways to leverage VAI to provide accessible information and resources to older adults and caregivers, support independence, or complement the broader care ecosystem through a set of connected devices. We summarize design implications for VAIs for informal aging care in Table 3.

**Table 2.** Design implications for informal VAI^a^ care systems.

Design implication	Ethical dimensions addressed	Suggestions
D1: encourage trust through transparency	Transparency, trust	Incorporate subtle opportunities for accessing information sources Help users gauge when AI^b^ is unsure by adding fact-checking features or the ability to flag information of uncertain origin
D2: prevent unauthorized access	Trust, privacy	Implement industry-standard security protocols to protect user data Be transparent about privacy policies at the start of the interaction Let users control the kind of information they share with companies and caregivers Leverage VAI capabilities (eg, voice biometrics) to automate system authorization processes Suggest alternatives to voice commands, such as text input
D3: provide personalization through context	Justice and fairness	Leverage contextual awareness such as environmental and user cues to deliver more relevant and dynamic responses (eg, gauging mood) and ensure more secure interactions (eg, verifying access)
D4: support autonomy through awareness	Freedom and autonomy	Incorporate clear and user-friendly features that let users easily override or disable AI suggestions whenever they want Enable personalization or automatically recognize and respond proactively to cues that indicate a need for human interaction
D5: provide flexibility through integrated support	Justice and fairness	Leverage voice input and output to provide accessible information and resources Leverage voice input to enhance older adults’ independence in managing self-care and well-being Leverage voice input and output for hands-free interaction with other devices that support care (eg, health education and demonstration materials, and wearables)

a VAI: voice-enabled artificial intelligence.

b AI: artificial intelligence.

### Limitations

This research includes some limitations. While we conducted some in-person studies, much of the work took place online to make it more convenient for our participants. Moving the studies online might have prevented some participants from engaging in the work. For example, those who have lower digital literacy or limited access to technology. This study also had more caregiver participants than older adult participants. Although we did see recurring themes within and between groups, our sample was also smaller overall. We also acknowledge that, because of the age similarities between our older adult participants and caregiving participants (one caregiving participant was aged younger than 55 years) and the flexibility of caregivers to also comment on their experiences as older adults, older caregiver perspectives may be overrepresented. Additionally, while we attempt to provide a rich description [46] of the participants and their experiences through demographic data and personas, because individuals’ care experiences vary, the participants’ experiences in our study may not transfer to all populations and contexts. There may be additional ethical values that did not emerge from our data, or that may not apply in different contexts.

### Conclusions

This study explores the experiences and challenges of older adults and informal family caregivers in managing informal care. Through participatory speculative design, we explore how future VAI technologies might support the caregiving process and address caregivers’ needs and concerns. We intentionally explore the ethical dimensions that participants value, and that may affect the potential adoption of VAI technology in informal care. Our findings provide insight into the need for VAI systems designed for the informal care context to balance efficiency and ethical values, supporting care processes while doing no harm.

## Supplementary material

10.2196/79740 Multimedia Appendix 1 List of additional tables and figures.

## Abbreviations

AI: artificial intelligence
HCI: human-computer interaction
RQ: research question
VAI: voice-enabled artificial intelligence
VUI: VUI
