# Supplementary material for: Exploring Expectations, Ethical Dimensions, and Values for Voice-Enabled AI Assistants That Support Older Adults and Informal Family Caregivers: Participatory Speculative Design Study
Source: JMIR Aging. 2026 Jun 16;9:e79740. doi: 10.2196/79740 (PMC13273013; doi:10.2196/79740)
Supplement: Multimedia Appendix 1 — List of additional tables and figures. [file aging-v9-e79740-s001.docx]

**Table S1.** Description of AI Ethical Dimensions [14] that emerged from workshop sessions.

| **Ethical Dimensions** | **Definition [14]** |
| --- | --- |
| Transparency | Transparency focuses on communication and disclosure efforts that explain and help people interpret AI actions. |
| Trust | Trust often has overlapping definitions with other dimensions including non-maleficence and responsibility and accountability. Trust in AI refers to a user’s ability to rely on and hold an AI system accountable. |
| Privacy | Privacy values emphasize the duty of AI systems to protect and secure personal data. |
| Justice and Fairness | Justice and Fairness involve actions to prevent unwanted bias and discrimination including focus on inclusion, equality, diversity, and accessibility. |
| Freedom & Autonomy | Freedom and autonomy relate to users’ feelings of choice, empowerment, and independence in interaction with an AI system. |

**Table S2.** High-level themes and example subcodes resulting from thematic analysis.

| **Theme** | **Description** | **Example Subcodes** |
| --- | --- | --- |
| Caregiving Experiences | Older adult and caregivers lived experiences of informal care. | Caregiving task, Caregiving values, Narratives of Experiences, Open Challenges within Caregiving |
| Opinions about VAIs for Caregiving | Ideas generated about how VAIs can or cannot support the caregiving experience | Older adult and caregiver envisioned VAI Uses, VAI concerns, Rationale/Benefits of VAI use, Tradeoffs of VAI use |
| Ethical Dimensions and Values | Ethical concerns discussed related to using and integrating VAIs in care | Transparency about health data use; Access and Personalization; Trusting Information from VAIs; Privacy and Confidentiality; Fairness and Inclusivity |

**Figure S1.** Examples of prompts used in design sessions to aid in discussions and role-playing to elicit ideas about Voice technologies that might support future informal caregiving efforts.


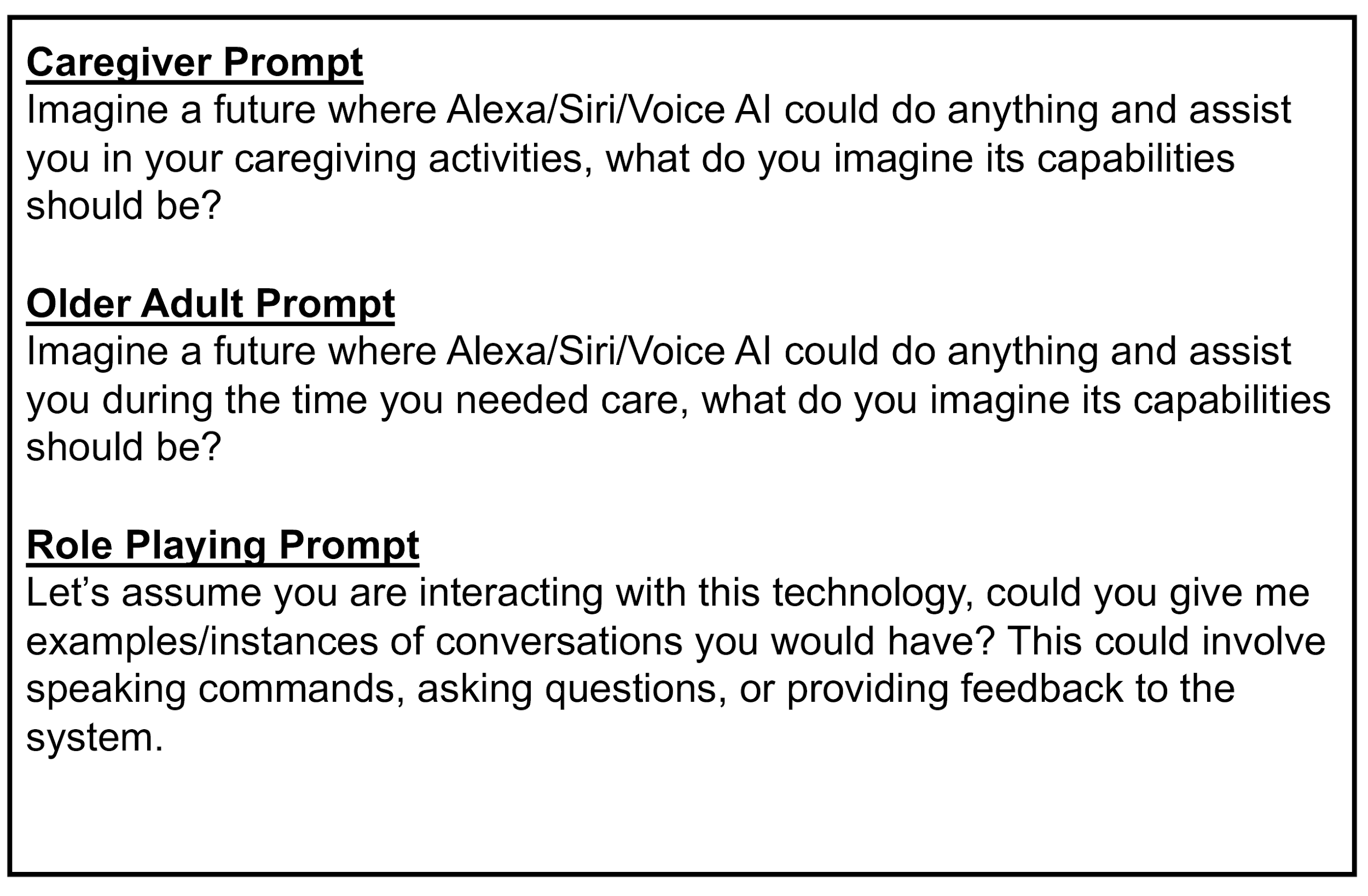


**Figure S2.** Two photos showing materials generated in workshops. The first is a whiteboard filled with yellow sticky notes capturing brainstorming ideas related to VAI technology and aging. The second is a collection of brainstorming materials spread out on a table.


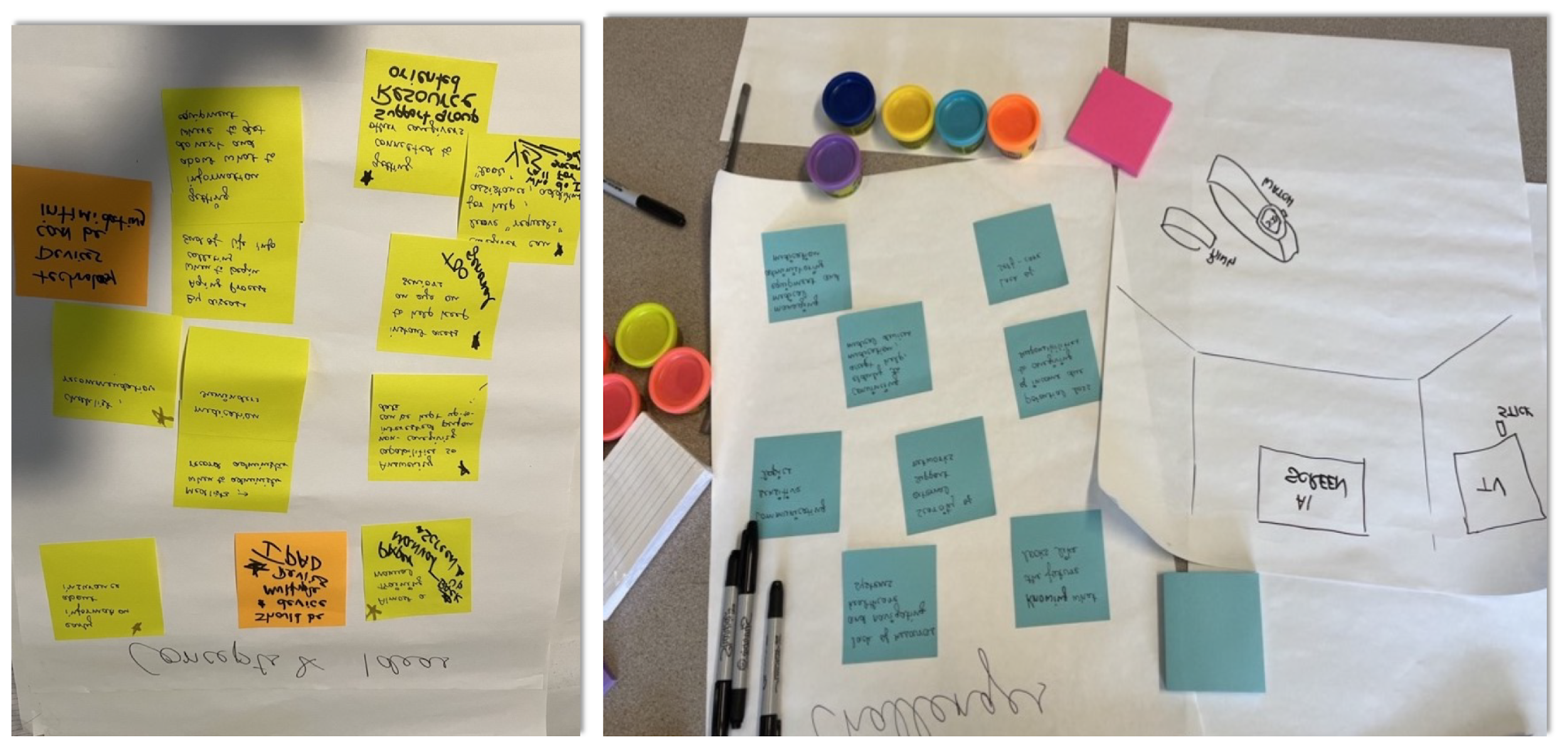


**Table S3.** List of caregiver ideas of how VAI might support their informal care processes.

| **Caregivers' Ideas for Voice Activated Support** | **Potential Value** |
| --- | --- |
| Voice-Activated information and resource requests | Timesaving |
| Predicting tasks and offering proactive help& Reducing stress | Reducing stress, Timesaving |
| Persuading older adults to accept help | Reducing stress, Improving relationship |
| Connecting to support groups | Reducing loneliness and overwhelming feelings |
| Controlling home equipment | Safety, Comfort, Independence |
| Appointment scheduling and management | Timesaving, Task management |
| Automatic stress recognition and support | Reducing loneliness and overwhelming feelings |
| Connecting with healthcare and emergency contacts | Reducing stress, Timesaving |
| Emergency assistance | Reducing stress |
| Emotional support and companionship | Reducing loneliness and overwhelming feelings |
| Recording messages in caregiver's absence | Continuity of care, Productivity |
| Encouraging self-care | Reducing stress |
| Troubleshooting by identifying patterns | Reducing stress, Timesaving |

**Table S4.** Older adults' ideas of how VAI might support their informal care processes.

| **Older Adults' Ideas for VAI Support** | **Potential Value** |
| --- | --- |
| Companionship ( dialogue, chat) | Reducing loneliness |
| Voice-activated information requests | Timesaving, Accessibility |
| Voice-activated entertainment | Timesaving, Reducing stress |
| Emergency assistance | Safety, Immediate help |
| Appointment reminders | Time management, Reliability/scheduling |
| Virtual companion | Reducing social isolation |

**Figure S3.** Hand-drawn sketch demonstrating interactions between an informal caregiver and VAI that proactively predicts mood and makes suggestions if stress is detected.


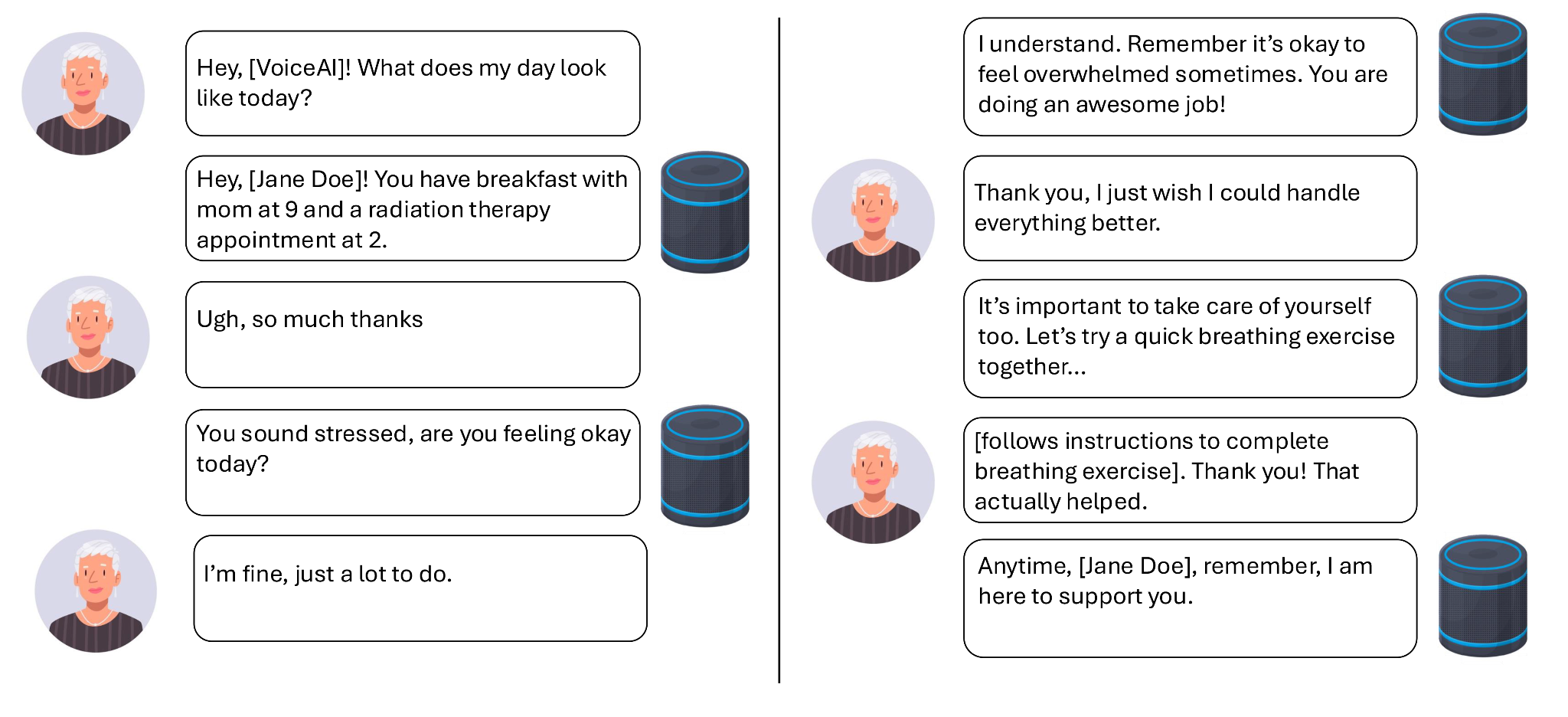


**Table S6.** An extended description of design implications and ethical dimensions.

| **Design Implication** | **Explanation** | **Ethical Dimensions Addressed** | **Suggestions** |
| --- | --- | --- | --- |
| D1: Encourage Trust Through Transparency | Incorporate features to help users trust and access the credibility of information received | Transparency, Trust | (1) incorporate subtle opportunities for accessing information sources  (2) help user gauge when AI is unsure by incorporating fact-checking mechanisms or the ability to flag information of uncertain origins |
| D2: Prevent Unauthorized Access | Consider dynamic privacy needs and offer multi-layered privacy controls. | Trust, Privacy | (1) implement industry-standard security protocols to safeguard user data  (2) be transparent about privacy policies in the beginning of the interaction  (3) allow users to control the type of information they share with companies and with caregivers  (4) leverage VAI capabilities (e.g., voice biometrics) to automate system authorization processes  (5) offer alternatives to voice commands, such as text input |
| D3: Provide Personalization Through Context | Leverage personalization to account for individual needs as well as contextual factors. | Justice and Fairness | (1) leverage contextual awareness such as environmental and user cues to provide more relevant and dynamic responses (e.g., gauging mood) and more secure interactions (e.g., verifying access) |
| D4: Support Autonomy Through Awareness | Foster a sense of agency and independence within the system by balancing assistance and user values. | Freedom and Autonomy | (1) incorporate clear and easy-to-use features that allow users to easily override or disable AI suggestions whenever desired  (2) allow personalization or alternatively proactively and automatically recognize and respond to cues that indicate a need for human interaction |
| D5: Provide Flexibility Through Integrated Support | Leverage voice abilities to support holistic support and care within the caregiving environment | Justice and Fairness | (1) leverage voice input and output to support accessible information and resources  (2) leverage voice input to support older adult independence in completing self-care and well-being  (3) leverage voice input and output for hands-free input when interacting with other devices that support care (e.g., health education and demonstration materials, wearables) |
